# A Novel Fragmentation-based Approach for Accurate Segmentation of Small-sized Brain Tumors in MRI Images

**DOI:** 10.2174/0115734056305784240821045425

**Published:** 2025-03-06

**Authors:** Mohd. Anjum, Sana Shahab, Shabir Ahmad, Taegkeun Whangbo

**Affiliations:** 1 Department of Computer Engineering, Aligarh Muslim University, Aligarh, India; 2 Department of Business Administration, College of Business Administration, Princess Nourah bint Abdulrahman University, PO Box 84428, Riyadh 11671, Saudi Arabia; 3 Department of Computer Engineering, College of IT Convergence, Gachon University, Seongnam 13120, Republic of Korea

**Keywords:** Artificial intelligence, Machine learning, Convolutional neural network, Feature extraction, Segmentation, Tumor detection

## Abstract

**Aims:**

In the dynamic landscape of healthcare, integrating Artificial Intelligence paradigms has become essential for sophisticated brain image analysis, especially in tumor detection. This research addresses the need for heightened learning precision in handling sensitive medical images by introducing the Fragmented Segment Detection Technique.

**Background:**

The ever-evolving healthcare landscape demands advanced methods for brain image analysis, particularly in detecting tumors. This study responds to this need by introducing the Feature Segmentation and Detection Technique (FSDT), a novel approach designed to identify brain tumors precisely using MRI images. The focus is on enhancing detection accuracy, even for diminutive tumors.

The primary objective of this study is to introduce and evaluate the efficacy of FSDT in identifying and sizing brain tumors through advanced medical image analysis. The proposed technique utilizes cross-section segmentation and pixel distribution analysis to improve detection accuracy, particularly in size-based tumor detection scenarios.

**Methods:**

The proposed technique commences by fragmenting the input through cross-section segmentation, enabling meticulous separation of pixel distribution in various sections. A Convolutional Neural Network then independently operates sequentially on the minimum and maximum representations. The segmented cross-section feature, exhibiting maximum accuracy, is employed in the neural network training process. Fine-tuning of the neural network optimizes feature distribution and pixel arrangements, specifically in consecutive size-based tumor detection scenarios.

**Results:**

The FSDT employs cross-sectional segmentation and pixel distribution analysis to enhance detection accuracy by leveraging a diverse dataset encompassing central nervous system CNS tumors. Comparative evaluations against existing methods, including ERV-Net, MRCNN, and ENet-B0, reveal FSDT's superiority in accuracy, training rate, analysis ratio, precision, recall, F1-score, and computational efficiency. The proposed technique demonstrates a remarkable 10.45% increase in accuracy, 14.12% in training rate, and a 10.78% reduction in analysis time.

**Conclusion:**

The proposed FSDT emerges as a promising solution for advancing the accurate identification and sizing of brain tumors through cutting-edge medical image analysis. The demonstrated improvements in accuracy, training rate, and analysis time showcase its potential for effective real-world healthcare applications.

## 1 INTRODUCTION

Examining brain tumors is an essential part of contemporary medicine, and many techniques are used to improve the precision of the diagnosis. Using machine learning (ML) and artificial intelligence (AI) to analyze brain images is a common technique. Depending on their size, location, and rate of growth, the growth in the brain can be either benign or cancerous and cause various symptoms. As such, identifying it is an essential responsibility for any healthcare facility. With the development of magnetic resonance imaging (MRI), the rate at which brain disorders are detected has significantly increased recently. MRI scans offer a detailed representation of the brain's structure and function, allowing for the acquisition of multi-angle and multi-modal images with minimal harm to the human body. Brain tumor recognition refers to identifying the presence of tumors in medical images such as MRI or computed tomography scans [1]. A radiologist can do this process manually or through computer-aided diagnosis systems, which use techniques such as image processing, imaging scans, M, artificial neural networks, and deep learning algorithms to detect and analyze tumors automatically [2]. MRI scans are a powerful tool for detecting brain tumors, providing detailed images of the brain's internal structure. These images identify important features and patterns of tumors, which are then used in the recognition and detection [3]. The presence and location of a brain tumor can be determined because of MRI scans' capacity to differentiate between various tissue types, including tumor tissue, normal brain tissue, and fluid-filled areas. In medical facilities, the MRI-based recognition approach is a commonly used technique [4].

Additionally, Magnetic Resonance Imaging (MRI) scans are non-invasive and do not use ionizing radiation, making them a safe imaging method for brain tumor. Brain tumor recognition methods classify the different types of brain tumors [5]. The accurate detection and segmentation of tumors are crucial for effective treatment. Artificial neural network (ANN) models are widely used to recognize brain tumors with MRI brain images to identify and distinguish the exact tissues and tumors in the brain, resulting in improved accuracy of brain tumor recognition [6]. Still, ANN faces limitations, such as blurred tissues in the boundaries and complex structure.

Hence, a level set method based on sparse constraint was applied to realize the tumor segmentation in MRI and accurately segment the tumor tissues [7]. This method weakens the influence of bright voxels within the tumor region, possibly reflecting in accuracy results. Therefore, an enhanced spatial attention network was employed to increase the accuracy of computed tomography with the feature extraction behaviour and the location information. The dependency on feature layers may impact the model degradation [8]. Due to this model, an ensemble approach was suggested to reduce the generalization errors of all models and make the final prediction with the multiple segmentation networks [9]. Each network is trained on the entire training phase of the dataset and impacts the region coverage of segmented tumors. The efficiency of segmentation algorithms is significantly impacted by various issues, including uneven image texture, noisy images, chaotic objects, occlusion, and others [10]. Accurately and rapidly segmenting brain tissue from tumors is a chronic difficulty. An updated dual-stream decoding convolutional neural network (CNN) structure is integrated with U-net to perform automated brain tumor segmentation on MR images [11] to improve brain tumor segmentation performance. The limitation is that it focused only on edge segmentation, not the entire tumor region. ML-based detection systems use different algorithms to analyze MRI images and identify specific patterns and features associated with certain conditions, such as brain tumors [12].

In the field of medical imaging, segmentation is an important process for the detection of brain tumors using ML algorithms. It involves dividing an MRI image into multiple parts, or segments, to accurately identify and separate the tumor from surrounding tissues [13]. Segmentation aims to eliminate noise and undesirable data from the MRI images to depict the boundaries and structure of the tumor accurately and train ML algorithms for identifying brain cancers in subsequent MRI scans [14]. The segmentation process distinguishes the region of interest (ROI) from the MRI images. Deep reinforcement learning algorithms are applied to extract important features and data from segmented MRI images [15]. Deep reinforcement learning is a subfield of ML that combines reinforcement learning with deep neural networks to solve problems such as brain tumor detection in medical imaging. These algorithms use image segmentation techniques and decision-making algorithms to analyze MRI images and identify ROI that may indicate the presence of a brain tumor [16].

The difficulty in obtaining accurate brain tumor detection, particularly for tiny lesions, drives the research motivation. Improvement is necessary because existing techniques don't provide the required accuracy for tiny lesions. The Fragmented Segment Detection Technique (FSDT) is an innovative approach that uses CNN for sequential analysis to close this gap. The goal is to advance medical image analysis by providing a more precise and effective way to identify brain tumors, ultimately leading to better diagnostic results and more effective healthcare. An important healthcare problem that this study aims to solve is the rapid and precise diagnosis of brain cancers using medical imaging, particularly Magnetic Resonance Imaging. The study discusses the challenge of identifying brain tumors in medical images, particularly magnetic resonance imaging scans. Instead of concentrating on segmentation or classification, the suggested method aims to enhance the efficiency and accuracy of brain tumor identification. The program improves the detection ratio by analyzing input MRI images, extracting characteristics, and using cross-sectional segmentation. Finding out whether and how big brain tumors are is the main objective. Minimizing uncertainty, increasing precision, and attaining a high detection ratio are the three primary goals highlighted by the authors. The suggested method combines pre-diagnosis with cross-sectional analysis and training with AI and ML procedures, specifically with the CNN model for feature learning and enhancement. The assessment metrics reflect the algorithm's performance in tackling the problem of brain tumor identification, which includes training rate, analysis ratio, recall, precision, analysis duration, and F1-score.

The objective is to increase the accuracy of brain tumor detection, especially concerning small-sized tumors using FSDT, addressing a critical gap in current medical imaging capabilities. The research focuses on greater learning precision in interpreting delicate medical images, which helps produce more accurate and subtle tumor detection. Learning precision measures how well a machine learning model consistently and accurately identifies and classes occurrences in a dataset. To detect tumors in the brain means how well the suggested FSDT learns and distinguishes characteristics linked to brain tumors. A high level of learning precision shows that the algorithm successfully finds and labels tumour-related areas in MRI scans. Accurate predictions directly influence patient outcomes, lowering the incidence of misdiagnoses and needless procedures, making this idea vital in medical applications. We have presented a novel method that prevents errors across duplicated segments and provides a thorough understanding of tumor existence, impact, and developmental stages: cross-section segmentation combined with pixel distribution analysis and CNN learning. The strategy is novel and distinct in integrating proactive mistake avoidance strategies to guarantee the dependability and resilience of the detection procedure.

This research makes significant contributions, namely:

1. FSDT is being introduced as a novel method to enhance the detection of brain tumors.

2. Enhancing the accuracy of brain tumor detection, especially for small-sized tumors, thereby addressing a crucial challenge in medical imaging.

3. Employing a sequential analysis methodology to prevent errors and optimize neural networks ultimately improves learning precision and aims to improve detection performance and reduce false positives and negatives.

The research paper comprised five sections. Section 1 provides an overview of the significance of brain tumor diagnosis and the role of MRI in medical imaging. Section 2 highlights the existing research in brain tumor segmentation and its limitations. Section 3 aims to present an FSDT for segmenting brain tumors from MRI images, which comprises fragmentation assessment and feature distribution using CNN. Section 4 evaluates the performance of the proposed method using metrics such as detection accuracy, training rate, analysis ratio, and analysis time and compares it with existing models. Finally, section 5 summarizes the contributions and the conclusion.

## 2 RELATED WORKS

In this segment, we provide an overview of the research undertaken on ML and deep learning methodologies aimed at identifying and categorizing brain tumors resulting from infections, as well as standard images.

### 2.1 Deep CNN Models

Shah *et al*. [17] proposed a deep CNN model based on EfficientNet-B0 (ENet-B0), which was modified and fine-tuned with extra layers to classify and detect brain tumors accurately and efficiently. The quality of the MRI images and the data size were increased for enhanced training through various filters and data augmentation techniques with an overall accuracy of 98.87%. The proposed method improves the efficiency of the diagnostic process and the accuracy of tumor identification. However, more advanced and effective deep CNN models have a chance of using segmentation that can be developed for brain tumor detection and classification with more efficient processing than the presented study.

Yu *et al*. [18] presented a new method for classifying brain diseases using an improved sparrow search algorithm to select the best image features from a pool of features and improve classification accuracy. The research outcomes showed that an improved sparrow search algorithm with a K-nearest neighbor removed 65.9% of useless features while achieving an accuracy rate of over 85%. These results indicate that the proposed approach improves the effectiveness of detecting brain diseases concerning other comparison algorithms and classifiers. The proposed model can be applied to other diseases and medical images. Segmentation can also be performed to extract features more accurately, but there is still limited exploration of advanced models.

Ahmad *et al*. [19] examined various transfer learning-based deep learning networks for brain tumor detection using traditional classifiers. These models were applied to labelled MRI datasets with normal and abnormal brain images. The performance of all combined models was evaluated in terms of accuracy, precision, recall, F1-score, Cohen's kappa, AUC, Jaccard, and Specificity, and the optimal model obtained an accuracy of 99.39% with a 10-fold cross-validation. Segmentation can also be performed to extract features more accurately, and extension in the dataset and high-speed GPU processing could enhance the accuracy and computational efficiency of the presented models.

Ottom *et al*. [20] developed a DL model and applied an augmented dataset to segment the brain tumor in 2D MRI scans. The DL algorithm segments the images based on features, and 2D images provide appropriate data for sorting and dividing into smaller parts. The proposed framework is built on skip connections, encoder-decoder architecture, and data amplification to enhance the intrinsic properties of a limited number of expertly defined tumors. Time and effort required ratios are reduced, increasing the systems' energy-efficiency range. The proposed model can also be extended for 3D images and other modalities.

### 2.2 Segmentation Models

Liang *et al*. [21] proposed an efficient transformer-based UNet for brain tumor segmentation. The segmentation method is mainly used to segment the pixel from the images, creating results that may be meaningful. This new strategy is an automatic MRI brain tumor segmentation process that provides feasible facts for the diagnosis process. The proposed technique applied a 3D parallel shifted window-based transformer to detect long-range contextual data for subsequent detections and provided increased effectiveness and reduced complexity. The recommended strategy decreases complexity, increasing the systems' effectiveness ratio by exploring lightweight approaches.

Sunsuhi *et al*. [22] developed an adaptive eroded deep CNN to segment the brain tumor images as three diseases: meningioma, glioma, and pituitary. The proposed adaptive eroded deep CNN was combined with Inception resnetV2 to classify meningioma, glioma, and pituitary brain diseases as benign or malignant. The adaptive eroded deep CNN finds the spatial information, while Inception resnetV2 handles the levels of information present in the input imagery data. The proposed model can be effectively used for segmentation and classification. The proposed model has a low error rate during diagnosis because of its high segmentation accuracy. It can serve as an efficient tool for physicians in medical imaging. However, segmentation and classification accuracy can be improved using various novel segmentation and filtering methods.

Ullah *et al*. [23] introduced an automatic brain tumor segmentation method using multi-scale residual attention-UNet. This technique processes three adjacent frames simultaneously to conserve the sequential information and uses multi-scale learning in a cascading method. It applied the adaptive ROI technique to improve the accuracy of the segmentation of core tumor regions. Furthermore, adaptive ROI is extracted based on characteristics and features, enhancing computational efficiency, productivity, and precision. Various post-processing techniques were applied to the output to enhance the overall performance of the introduced model, and integration with supervised DL models was suggested to avoid huge time consumption.

Zhou *et al*. [24] developed an efficient 3D residual neural network (ERV-Net) for brain tumor segmentation. The proposed method is lightweight, needing lower computational complexity and memory usage by incorporating 3D ShuffleNetV2 as the encoder, a residual block decoder, and a fusion loss function. A post-processing step was also introduced to enhance the segmentation results. Furthermore, the proposed strategy effectively reduces the segmentation latency, which enhances other processes' efficiency. In this 3D method, more lightweight approaches can be incorporated to decrease the number of parameters in the decoder, and the number of layers can be increased in the encoder to determine the larger receptive field. These enhancements will make the network more lightweight and efficient.

### 2.3 Multi-modal Image Segmentation

Pei *et al*. [25] developed two new models based on two approaches, feature fusion and joint label fusion, to predict brain tumor segmentation in longitudinal multi-model MRI images. The proposed method predicts information from both high and low-resolution MRI images. A random forest algorithm is used here that extracts the textural and functional features from the images. It also detects the important characteristics and patterns of brain tumors. The suggested approach's efficiency in image segmentation improves the systems' effectiveness.

Sun *et al*. [26] presented a 3D, fully CNN-based image segmentation for the brain tumor detection process for multi-model MRI images. The proposed model comprised multiple path 3D CNN to extract the features of scales from the input images and fuse them to transpose convolutional layers. The experimental result showed that this multipath architecture was more effective in segmentation than the signal path model with different stages, but it cost more time. The suggested DL technique for segmenting 3D MRI images is a powerful tool providing useful information for detecting and diagnosing brain tumours. Further exploration of segmentation accuracy.

Masood *et al*. [27] developed a new mask recurrent CNN (MRCNN)-based image segmentation for the brain tumor localization process. A binary classification technique was implemented to classify the features based on certain conditions and functions and predict the image regions as tumor or non-tumor. ROI and patterns were predicted and segmented to reduce the complexity of the brain tumor detection process. The results demonstrate the effectiveness of the proposed method in precisely separating the tumor region, serving as a useful tool for diagnosis. The suggested technique improves the accuracy rate over state-of-the-art approaches that produce necessary data for the brain tumor diagnosis process. The proposed method can be extended to classify different types of tumors.

Xiong *et al*. [28] proposed a field-programmable gate array-based accelerator model to map a specific neural network for brain tumor image segmentation. The neural network model was optimized through multiple training for a reduced number of model parameters obtained from combining batch normalization layers. The optimized network reduced the computational requirement for fast and efficient brain tumor segmentation, focusing on maintaining high accuracy while reducing power consumption. This approach presents a novel direction for automated and remote diagnosis of brain tumors. The MRI scans are highly sparse, with an average of 70% sparsity. Additional research on sparse MRI images is lacking. Utilizing this sparsity in the design of algorithms or hardware implementation could reduce the time spent on computing invalid data and improve performance.

Islam *et al*. [29] introduced a deformable snake model for brain tumor segmentation in medical images. The proposed model employed the parametric active contour model. The standard parametric active contour model requires manually initialized contour points to start computation, which is time-consuming. The proposed model mitigated this issue by automatically initializing at least three contour points in the ROI. This leads to improved and efficient segmentation results, as the proposed model can determine the initial contour points without manual input and begin the deformable process. The novel approach enhances the performance and efficiency range in the brain tumor segmentation process but is still limited to fewer tumor sizes in the MRI scans.

Iqbal *et al*. [30] proposed an automatic brain tumor segmentation using a super pixel-based approach with enhanced tuned model parameter values. MRI inputs produce important information for detecting and segmentation in this approach. The proposed segmentation method identified the textural, functional, and factual features from the superpixel images that can speed up the computing procedure. This novel model improves the performance and feasibility ratio of the brain tumor segmentation process.

Amran Hossain *et al*. [ 31 ] suggested the lightweight segmentation model called MicrowaveSegNet (MSegNet) for brain tumor segmentation using BrainImageNet (BINet) model to classify the reconstructed microwave (RMW) images. Our sensors-based microwave brain imaging (SMBI) system first collected 300 RMW brain image samples to establish a baseline dataset. Next, 6,000 training photos were created for each fold using image pre-processing and augmentation methods. This was done for a 5-fold cross-validation. Subsequently, to validate MSegNet and BINet's performance, they were contrasted with cutting-edge segmentation and classification models. Regarding tumor segmentation, MSegNet has attained an IoU score of 86.02% and a Dice score of 93.10%.

Dinesh Babu and Senthil Singh [ 32 ] recommended the Level Based Learning Model for Brain Tumor Segmentation. In their early stages, tumors are often less noticeable and more spherical, making them easy targets for Convolutional Neural Network (CNN) algorithms. This proposal aims to provide a comprehensive model that can detect cancer in its early stages and segment tumors regardless of their size or shape. The shortcomings of earlier models are eliminated, and the quality of smaller tumor cells and forms is enhanced when the convolution process is dilated, allowing feature extraction to cope with a multi-scale goal and level-based learning. Highlighting the detection of small-scale tumor development, the feature reconstruction operations are encapsulated by the level-based learning technique. Compared to hierarchical methods, picture segmentation is executed more accurately, leading to improved detection.

D. Ramya and C. Lakshmi [33] introduced the Hybrid Heuristic-Aided Multi-scale Self-Guided Attention Mechanism for Brain Tumor Segmentation. A new 3D brain tumor segmentation model based on hybrid heuristic development is suggested to solve these difficult elements. To begin, pictures of the brain are taken from well-known benchmark datasets. Limited Adaptive Histogram Equalization (CLAHE) is an adaptive method to pre-process the acquired brain pictures. The experimental study aims to compare the constructed framework using various segmentation approaches to determine its efficiency.

Junding Sun *et al*. [34] presented the Multi-view attention and multi-scale feature interaction for brain tumor segmentation. To address global and local issues, this method suggests a multi-view attention mechanism that considers location, content, and channel. The target region may be more accurately located, and features can be better represented in lesion regions thanks to this technique. Also, to encourage interaction across dimensions, the author creates a multi-scale feature interaction module that picks and chooses useful information from several receptive fields of different sizes. Because of this, the approach allows for the accurate segmentation of the border contours of various lump types. The experimental findings show that the suggested technique achieves better accuracy in segmenting brain tumors compared to other methods.

Over the years, numerous research works have been undertaken to apply image processing techniques for computerized medical image analysis. Existing methods, such as ERV-Net, MRCNN, and ENet-BO, have contributed significantly to tumor detection, yet they face challenges in achieving optimal accuracy and efficiency. Brain tumor detection methods are diverse, with ERV-Net, MRCNN, and ENet-B0 being key innovations. ERV-Net provides a foundational understanding, achieving a moderate accuracy of 63.865%. MRCNN improves accuracy with a notable accuracy of 70.68%. ENet-B0 builds upon prior models, showcasing a significant accuracy boost to 82.14%. FSDT, a new approach, introduces cross-sectional segmentation, optimizes the training rate, and enhances the analysis ratio. FSDT places a premium on precision, achieving a 12.56% increase in precision and reducing analysis time by 10.78%. FSDT emerges as a robust and innovative approach, showcasing significant advancements in accuracy and computational efficiency for real-world healthcare applications. Hence, this research discusses the need for increased precision in brain tumor identification, focusing on small-sized tumors. While existing approaches use neural networks and cross-sectional segmentation, they are not entirely successful in precisely recognizing and classifying tumors according to size. The FSDT is a cutting-edge method intended to improve detection ratios, especially for small-sized tumors, to close this gap. Using a CNN to optimize the extraction of features and pixel distribution, FSDT sequentially analyzes fragmented cross-sections.

## 3 METHOD

### 3.1 Proposed Fragmented Segment Detection Technique

The proposed FSDT is designed to identify the accuracy of tumors and their size in a human body and is analyzed through MRI image fragmentation and cross-sectional segmentation. MRI imaging of various brain tumor types may reveal distinctive features; hence, the proposed FSDT technique is employed to identify the tumor types, such as glioma, meningioma, and pituitary tumors. In radical brain tumors recognizing scenarios, sensitive medical images are handled and analyzed for improving precision. The medical image contains shape, texture, and content features to collect and analyze information stored from the pre-diagnosis. The cross-sectional segmentation of large and small-sized tumors is observed from the input MRI image to detect the fragmented information in a human brain. The research assertion raises concerns regarding the potential limitations of human accuracy in analyzing MRI slices. It shows that certain healthcare providers prefer manual over machine-based procedures because they trust their experience. When experts recognize the drawbacks of human interpretation, it highlights the potential benefits of incorporating AI-driven techniques, such as the FSDT. The objective is to increase precision, reduce subjectivity, and provide medical professionals with a solid support system for examining brain tumor images. The given input image is fragmented using cross-sectional segmentation to identify the tumors and analyze brain images employed by AI in healthcare. The proposed FSDT identifies the accuracy of tumors, and their size is analyzed using the cross-section segmentation of the input MRI images. The overview of FSDT is presented in Fig. (**1**).

The detailed representation of the research idea presents the FSDT, which uses MRI image analysis to detect brain tumors accurately. FSDT uses ML and AI to increase the accuracy of tumor identification and size estimation. Using cross-section segmentation, the input MRI picture *MRI_image__N_* from the machine is broken up, and the pixel distribution for each region is then examined. Feature extraction associated with tumor size is the aim of CNN. The essay emphasizes brain tumors, highlighting the significance of managing delicate medical pictures in the healthcare industry. Next, FSDT is split into two sections: feature distribution and fragmentation assessment.

### 3.2 Fragmentation Assessment

ML is responsible for observing and analyzing brain images from the healthcare field. The pre-processing is performed for brain image analysis based on feature extraction. *f_x_* and removes noise *n_o_* occurrence in the input MRI images. The features such as image edge *E_g_*, horizontal contrast*C_H_* and vertical contrast *C_V_* are extracted from the input image. The MRI image output *MRI_image__∆_* is required to compute the gradient magnitude of each pixel. Assume the input image can be fragmented to leverage the tumor detection ratio using cross-sectional segmentation. In this brain image analysis instance, the fragmented segment, as identified (*F_seg_*) in the human brain is expressed as:

**Table:** 

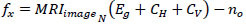	(1)

Such that,

**Table:** 

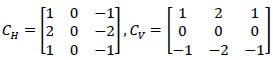	(2)

**Table:** 

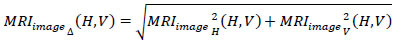	(3)

**Table:** 

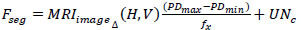	(4)

**Table:** 

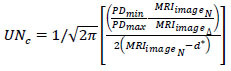	(5)

From Eqs. (**1**-**5**), *MRI_image__N_* is the present input MRI image; therefore *MRI_image__N_* ϵ *MRI_image__∆_*. The variable and *PD_min_* means the maximum and minimum pixel distribution observed in different sections. The variable *UN_c_* and d* represents the uncertainty and pre-diagnosis information identified. The fragmentation and sectional illustrations are shown in Fig. (**2**).

#### 3.2.1 Pre-processing Steps

**Fig. (2):**
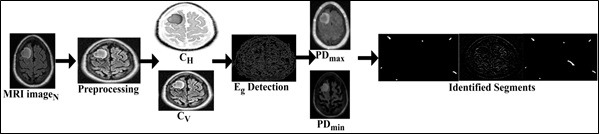
Fragmentation and sectional illustration.

The input is pre-processed (*i.e*.) the fundamental grey-scale conversion is performed from which *C_H_* and *C_v_* distributions are classified. This is performed to evade *n_o_* in any pixel concentration. Pursued by the contrast, the edges before sectional representation are detected. Such detection identifies the chances for sparse and dense pixel distribution. This is required for reducing errors in segmentation due to overlapping and *UN_C_* (Refer to Fig. **2**). The uncertainty of tumors in the human brain is represented as the number of swelling or tumors observed from the image at different sections *s*. There are some cases of noises present in the *MRI_images__N_* due to feature extraction and analysis problems. Therefore, these problems impact the given *MRI_images__N_* analysis at different sections, for which the pixel distribution checking is performed as:

**Table:** 

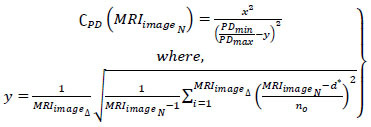	(6)

In Eq. (**6**), the checking of pixel distribution in *MRI_images__N_* is separated for the various sections and identifies the maximum pixel representation (*x*) and the minimum pixel representation (*y*). Here, the minimum pixel representation is a normalized measure, whereas the maximum pixel representation is the uncertain measure for identifying tumors' presence and size in the brain.

#### 3.2.2 Fragmentation Identification

The terms “minimum” and “maximum representations” most likely relate to the pixel distribution values discovered by MRI image processing. The pixel distribution represents the difference in pixel intensity between various image regions. In a given segment, the maximum representation *PD_max_* may represent the greatest observed pixel value, while the minimum *PD_min_* represents the lowest. Eq. (**7**) essentially uses the range of pixel intensities, minimum to maximum, inside a particular segment of the input MRI image to evaluate the uncertainty of tumor detection. This uncertainty metric is used by the neural network and its subsequent processing to detect and classify brain cancers. PD_max_ represents the maximum pixel intensity in that area, which correlates to the brightest pixel. PD_min_ indicates the pixel intensity that is the lowest in a particular area or section of the MRI picture. This lowest value indicates the tumor area's dimmest pixel and acts as a baseline.

Based on *MRI_images__N_* and C_*PD*_(*MRI_images__N_*), the sequential analysis of minimum and maximum pixel representation operated by the CNN is estimated as:

**Table:** 

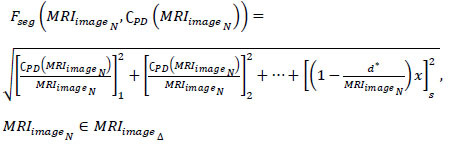	(7)

Eq. (**7**) evaluates the uncertainty for minimum and maximum representation sequentially until *M* is active in processing brain images from the healthcare field. AI and ML will handle sensitive medical images until the healthcare field requires the service of tumor detection. The above sequence of minimum and maximum pixel representation is analyzed using CNN. In image processing, the observed information from the input MRI images is to be converted into text that must be distributed to the medical field to identify the accuracy of tumors, precise location, and size to improve the detection ratio. Besides, the pixel representation is to be instantaneous to meet the radical demands of handling sensitive medical images. Therefore, AI connected ML and the neural network used for *F_seg_* assessment in this article. The output of the CNN learning is to identify and separate the unsynchronized pixel distributions through *MRI_images__N_* analysis and pre-diagnosis-based training. In this learning, the first step is to sample *MRI_images__N_*, if *F_seg_* is observed. The segmented cross-section features of achieving 
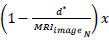
 previous assessment and the precise output for classifying the pixel representations using CNN. For this purpose, two replicated segments of *MRI_images__N_* at different sections. Assume *P* and *Q* are feet as the input for the ML paradigm. For *F_seg_* the sections are modelled as per Eqs. (**8a** and **8b**).

**Table:** 

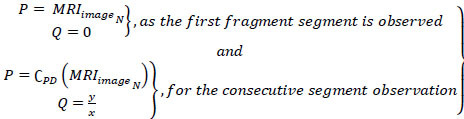	(8a)

Such that,

**Table:** 

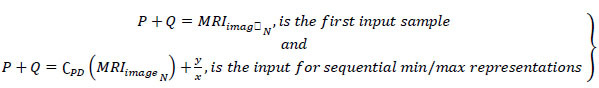	(8b)

Where the ML assessment initiates the sequential minimum and maximum representations with the first training processed as*MRI_images__N_*. This input image identifies accurate tumor occurrence; if a fragmented segment is observed, the pixel distribution is separated into different sections.

#### 3.2.3 Uncertainty and Pre-diagnosis using CNN Learning

In CNN, the consecutive minimum and maximum pixel representation of 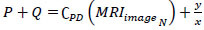 is addressed to remove all the uncertainty in tumor detection. The pixel minimal-maximal description *PD* is presented in Fig. (**3**).

**Fig. (3):**
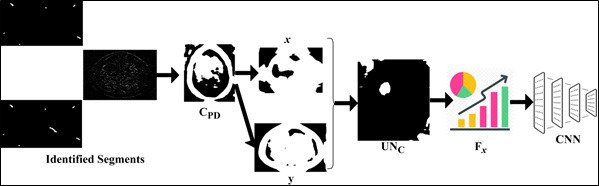
Pixel minimal-maximal description *PD*.

The representation follows (*x*, *y*) ϵ *C_PD_* such that the identified segments are validated for *P*, *Q* *P + Q*. The case where *Q = 0* is alone discarded and then the representation failing case is declared as *UN_C_*. Post this *UN_C_*, the extraction is performed across various *C_H_* and *C_V_* (independently). In this process, the minimum representation is monitored alone to prevent *Q* = 0 detections (Refer to Fig. **3**). CNN consists of two-pixel representations, min and max, followed by the image output. The sequential analysis of tumors presence and its impacts identified by the AI and ML function *MF* is represented in Eqs. (**9** and **10**).

**Table:** 

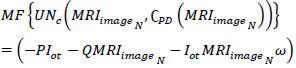	(9)

**Table:** 

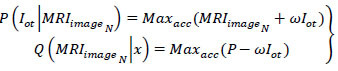	(10)

Where, *I_ot_* is the segmented cross-section features output, and *ω* is the tumor size observed by the machine from the *MRI_images__N_*. The maximum accuracy *Max_acc_* of segmented cross-section, features are used for performing training. In the above Equation, the two replicated segments of *P*(*I_ot_*|*MRI_images__N_*) and *Q*(*MRI_images__N_*|*x*) with maximum accuracy is used for satisfying the condition *MF*{*UN_C_*(*MRI_images__N_*, C*PD*(*MRI_images__N_*))}. As per the condition *P*(*I_ot_*|*MRI_images__N_*) and *Q*(*MRI_images__N_*|*x*), the given input MRI image fragmented assessment satisfies *x* and C*PD*(*MRI_images__N_*) and produces output *MF*{*UN_C_*(*MRI_images__N_*, C*PD*(*MRI_images__N_*))} at its nearest possible segment detection. Therefore, the minimum and maximum representations sequence lies between two replicated segments in different sections. The CNN functions are presented in Fig. (**4**).

**Fig. (4):**
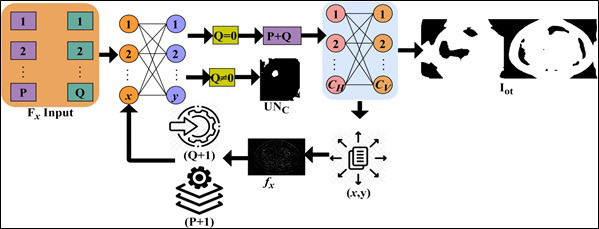
Convolutional neural network functions.

The neural network process distinguishes *Q* = 0 and *Q* 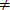 0 for various (*P* + *Q*) and 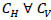 combinations. The combinations represent the pixel distribution (Even) for identifying *I*_*ot*_. If *I*_*ot*_ is valid, then maximum accuracy is attained, and therefore the distribution (same, except *Q* = 0 is used for *f_x_* and training. More specifically, the *f_x_* 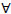 *x* and *y* are used for (*P* + 1) and (*Q* + 1) (same) for training (Fig. **4**). From here, pixel representation segmentation is initiated to identify tumors in the brain accurately. This segmentation represents the change in pixel arrangements and tunes its feature distribution using CNN. Based on this, the feature distribution based on segment detection is explained in the next section.

Image analysis represents an area where Convolutional Neural Networks shine. CNNs are specifically built to handle structured grid data. Convolutional layers in a CNN design streamline spatial dimensions, pooling layers extract features from MRI images, and fully connected layers map these data to the output. Rectified Linear Units (ReLU) and other activation functions bring non-linearity to the network, which helps it understand complicated mappings and spot nuanced patterns. Loss functions are problem-specific; for example, binary classification tasks like tumor detection often employ cross-entropy loss, especially binary cross-entropy. This study uses more advanced optimizers, such as Adam, with adaptive learning rates, rather than the more conventional Stochastic Gradient Descent (SGD), while training CNNs. The loss and learning curves demonstrate the model's ability to minimize the disparity between predicted and actual values. The learning curve also shows how the model's performance has changed across the training and validation sets. Ideal learning occurs when the two curves converge, meaning neither overfitting nor underfitting occurred.

### 3.3 Feature Distribution

In the feature distribution process, the AI-based MRI image analysis is processed by ML to identify the tumor's presence in the brain. Sequential minimum and maximum representation of pixels from the input image is analyzed with *MF*{*UN_C_*(*MRI_images__N_*, C*PD*(*MRI_images__N_*))} condition respectively.

#### 3.3.1 AI-based MRI Image Analysis

Assume that the initial feature distribution with maximum accuracy *Max_acc_* = 0 and pixel arrangements in consecutive size-based tumor detection using a neural network. If the condition *Max_acc_* = 1, then the segment detection is processed with maximum accuracy and used to identify accurate tumors and their size in the brain. This sequential analysis relies on ω and *MF*{*UN_C_*(*MRI_images__N_*, C*PD*(*MRI_images__N_*))} such that the probability of feature distribution (*ρ_FD_*) is computed as:

**Table:** 

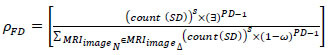	(11)

Eq. (**11**) estimates the probability of segment detection *SD* with maximum accuracy. It is used to ensure that ML is powered to identify the reform *r* or cure of a tumor in the brain. If 
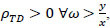
 then the count of pixel distribution is incremented by 1, which means in the input image tumor is identified; otherwise tumor is not detected in the input image. The variable 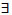 means the estimation of the changes in pixel arrangements between 0 (cured) and 1 (reform), and this feature is computed as:

**Table:** 

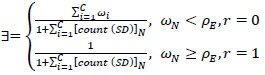	(12)

In Eq. (**12**), the changes in pixel arrangements are compared with previous images and then computed segment detection following all the sequential input images *C*. This computation of feature distribution with minimum accuracy results in an unsynchronized machine-learning process due to the probability of error occurrence *ρ_E_* in sequential analysis. With this analysis, the output of feature distribution with minimum and maximum accuracy (*δ*) in different sections is analyzed using *MF*{*UN_C_*(*MRI_images__N_*, C*PD*(*MRI_images__N_*))} and ω is expressed in Eq. (**13**).

**Table:** 

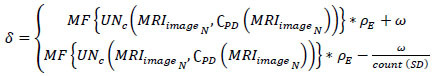	(13)

#### 3.3.2 Pixel Distribution

Similarly, the pixel distribution for the condition *MF*{*UN_C_*(*MRI_images__N_*, C*PD*(*MRI_images__N_*))} ϵ [*PD_min_*,-2*PD_max_*] is represented in Eq. (**14**).

**Table:** 

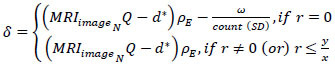	(14)

In the above Equation, the sequential pixel representation analysis identifies the tumor reformation and sizes through MRI images. The accurate detection process is illustrated in Fig. (**5**).

**Fig. (5):**
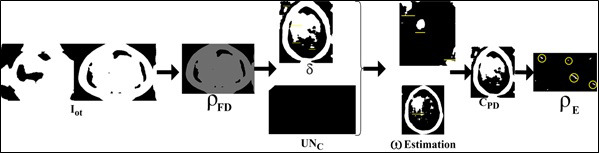
Accurate detection process.

The *ω* estimation requires *UN_C_* differentiation from *δ* using *ρ_FD_*. This*ρ_FD_* is observed from *I_ot_* of different sizes. The (*x*, *y*) are appropriate for *C_V_* and *C_H_* accordingly for preventing *ρ_E_*. However, they occurred *ρ_E_* is reduced using the *Q* = 0 (or) *Q* 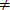 0 condition check (refer to Fig. **5**). The accuracy of tumors is detected using *F_seg_* and δ, the ML and AI in the healthcare field is 24 x 7 processing. In the brain image analysis, [*PD_min_*,-2*PD_max_*] is the maximum pixel representation of identified images whereas 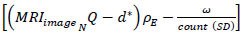 is the minimum pixel representation of identified images. The segregation of minimum pixel distribution helps improve the accuracy of learning precision.

### 3.4 Integrated Algorithm for FSDT in Brain Tumor Detection

**Step 1: **Input MRI Image (*MRI_image_N__* //Input

**Step 2: ** MRI_image_∆__= ComputeGradientMagnitude(*MRI_image_N__*, H, V) //Pre-processing E_g_, C_H_, C_V_ = ExtractFeatures(MRI_image_N__)

**Step 3: S**egments=CrossSectionalSegmentation(MRI_image_N__) //Fragmentation Assessment for each segment in segments:

PD_max_, PD_min_=CalcualatePixelDistribution(segment)

UN_c_=CalculateUncertainty(PD_max_, PD_min_)

**Step 4: **InitializeNeuralNetwork() // Learning process for each segment in segments:

TrainNeuralNetwork(segment, PD_max_, PD_min_)

**Step 5:** *ρ_FD_*=assessFeatureDistribution(MRI_images__N_, *ω*, Un_c_) //Feature Distribution

*ρ_E_*=ComputeProbabilitySegmentDetection(*ρ_FD_*

**Step 6: **OutputDetectionResult(*ρ_E_*)

The basic procedures of the FSDT, intended to detect brain tumors, are contained in the integrated algorithm called pseudocode. The input MRI picture is first pre-processed, where pertinent characteristics and gradient magnitude are calculated. The next step is fragmentation evaluation, which entails segmenting the image, calculating each section's lowest and greatest pixel representations, and evaluating the degree of uncertainty. Next, using CNN approaches, the learning process is started by establishing a neural network and then conditioning it separately for its maximum and minimum representations. The neural network examines each segment to determine whether a tumor is there, how big it is, and what stage it is in. The distribution of features is evaluated, considering pixel arrangements and uncertainties. Finally, the algorithm calculates the segment detection probability based on the highest level of precision, producing the final result that indicates the tumor's existence, size, and stage of development. The FSDT algorithm is briefly represented by this pseudocode, which offers an organized summary of its main features concerning brain tumor identification.

## 4 RESULTS AND DISCUSSION

The performance assessment uses the dataset from [35], which accounts for all primary Central Nervous System (CNS) tumors.

### 4.1 Dataset Description

The mentioned dataset is classified with the types of brain tumors like benign tumor, malignant, glioma and pituitary tumor. Based on the location of the swelling, these tumors can be categorized. A brain tumour is one of the more severe diseases affecting children and adults. 85 to 90% of all primary CNS tumors are brain tumors. About 11,700 patients are given a brain tumor diagnosis each year. For those who have a malignant brain or CNS tumor, the survival rate after five years is roughly 34% for males and 36% for women. MRI is the most reliable method for finding brain tumors. The scans provide an enormous amount of picture data. The radiologist examines these pictures. The sizes and locations of the brain tumor(s) are highly aberrant, and a qualified neurosurgeon is also necessary for MRI analysis. From this dataset, 3200+ MRI inputs are used for training the neural network under 800 iterations for *P* and *Q* independently. The maximum number of features extracted is 17, and the segments are 10 for a single training input; this is pursued unanimously. A sample segmentation scale for input due to the impact of (*x*, *y*), and *C_H_* and *C_V_* is presented in Table **1**. Databases created for classification are frequently modified or repurposed for segmentation tasks in medical image analysis. This is because these datasets contain labelled data regarding the presence and types of tumors. Segmentation activities, which include finding and defining the borders of structures or areas of interest within an image, can benefit from this data. Models can be trained to recognize tumor types using a classification dataset. These models can subsequently be used for segmentation tasks, which seek to delineate tumor regions accurately. Utilizing a dataset originally intended for a particular use in another call for meticulous deliberation and comprehensive assessment of the model's segmentation performance.

In Table **1**, the *C_H_* and *C_V_* for the varying segments are analyzed. The segment detection varies due to *C_PD_* and *FD* using *f_x_*. However, the (*x*, *y*) the combination is required for suppressing *Q* = 0; the *UN_c_* is reduced for consecutive cases. Using this process, the *I_ot_* is extracted for *ρ_E_* suppression. Therefore, variations are abnormal 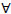 *Q* = 0 observed in *C*_H_. After this reduction, training for the same state is obtained to reduce error (train, best, validation). Variables such as *C_PD_*, *FD*, (*x*, *y*) and *Q* are presumably considered in the table's examination of pixel distributions and features across categories. Decreases in *UN_c_* for successive cases point to a method for improving the model's accuracy. Additional evidence of attempts to reduce uncertainty is the extraction of *I_ot_* for *ρ_E_* suppression. For *Q* = 0 cases, the unusual *C_H_* changes can represent the model's reaction to specific inputs.

### 4.2 Comparative Analysis

This study strives to assess the efficacy of the Flexible Segmentation and Detection Technique in detecting brain tumors by comparing it to prominent algorithms in the field. In this comparison, ERV-Net [24], MRCNN [27], and ENet-B0 [17] are considered foundational, as they each contribute unique methodologies and insights. ENet-B0 demonstrates efficiency in a small architecture, ERV-Net incorporates new methods, and MRCNN emphasizes careful region-based analysis. By delving into the details of these algorithms, we can learn more about the varied approaches to detecting brain tumors and set a standard for FSDT. This comparative analysis sheds light on FSDT's innovative contributions to the area and improves our understanding of its strengths.

### 4.3 Evaluation Metrics

This study assesses the efficacy of each algorithm in detecting brain tumors using thorough algorithmic evaluation measures. These measurements include accuracy in detection, training rate, analysis ratio, accuracy, recall (sensitivity), analysis duration, and F1-score. Detection accuracy is a metric for how MRI images can identify tumors. In iterative training, the training rate measures how quickly and efficiently the system learns new material. An algorithm's analysis ratio indicates its ability to eliminate ambiguity and produce reliable outcomes. Reducing the number of false positives and providing accurate results are the primary goals of precision. The computational efficiency of the method is measured by analysis time, which considers processing time considerations for both segments and features. The accuracy of the system in detecting cancers, which is critical for prompt diagnosis and treatment, is assessed by recall (sensitivity). The F1 score gives a complete view of the algorithm's capabilities by providing a detailed and all-encompassing metric for evaluating the model's performance.

In medical imaging, reliable diagnostic results depend on the model's detection accuracy. The accuracy with which tumors may be identified using input MRI scans is a metric. A more rapid learning process, as indicated by a higher training rate, speeds up the deployment of reliable tumor detection models. The analysis ratio measures how well the algorithm performs when analyzing brain images to minimize uncertainty. Precision evaluates how well tumours are detected, focusing on reducing the number of false positives. The computational efficiency of the algorithm is measured by its analysis time; a lower duration indicates efficient processing for real-time applications. A system's recall (sensitivity) measures how much it can detect malignancies in real-time, which is important for prompt diagnosis and treatment. The F1-Score gives a complete picture of the model's accuracy by combining recall and precision. A balanced model with a high F1-Score detects targets accurately while producing a few false negatives.

#### 4.3.1 Detection Accuracy

In Fig. (**6**), the accuracy of tumor detection based on observing a tumor or swelling in the human brain is analyzed through input MRI images for extracting some features for improving the detection ratio. The detection accuracy can be calculated using the given formula:

**Table:** 

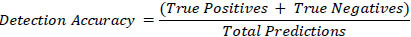

The extracted features are fragmented through pre-diagnosis using cross-sectional segmentation, and the pixel distribution is separated for the different sections. This proposed technique is processed to identify the uncertainty factor and errors observed from the input image analyzed by ML. The fragmentation and feature distribution are performed to identify the tumor presence and size, and the AI and ML sequentially analyze minimum and maximum pixel representation. The errors identified through CNN learning for improving brain tumor detection ratio. If the segmented cross-section features with maximum accuracy are used, then training is processed by the learning process. The CNN process can train and upgrade the minimum accuracy features for different sections, preventing analysis time. Therefore, the learning can be classified based on the minimum and maximum pixel distribution to achieve a high tumor detection ratio until a new section is trained.

#### 4.3.2 Training Rate

**Fig. (7):**
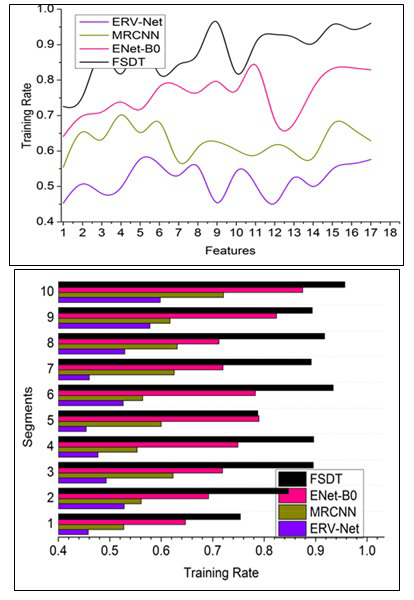
Training rate comparisons.

Fig. (**7**) shows training rate comparisons for the proposed FSDT with other models. This proposed FSDT for detecting tumors, and their size in the brain achieves a high training rate through pre-diagnosis. Training for generating new sections relies on brain image analysis at different sections (refer to Fig. **7**). The segmentation process of pixel representation is observed to identify changes in tumor size and reform through training. CNN is classified into two replicated segments for error occurrence identification. The fragmentation and segmentation of the individual brain image easily identify tumor occurrence for precise diagnosis through ML. The tumor developed in the brain is observed through input image and pre-diagnosis using feature distribution, and pixel arrangement computation is performed to extract the features. The tumor detection is performed using the CNN process, and training is used to reduce the analysis time for successful segment detection. Therefore, the pixel representation classification and its feature distribution outputs are used to improve the individual image's fragmentation. Hence, the training rate is high when separating the pixel representation.

#### 4.3.3 Analysis Ratio

This proposed technique achieves high brain image analysis computation through pre-diagnosis and fragmented segment identification for detecting the accuracy of tumors in the human brain and reducing uncertainty factors, as depicted in Fig. (**8**). The accurate tumor and tumor detection are aided in reducing the reform using pixel representation identification for tuning feature distribution. Using the given formula, the analysis ratio can be calculated.

**Table:** 

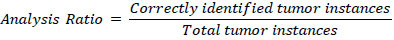

The sequential minimum and maximum pixel representation are observed through the neural network for successive tumor presence detection. The inaccuracy in tumor detection is mitigated through segment detection and feature distribution. Different training is processed to reduce the tumor size so that the input MRI image can be fragmented to leverage the precise tumor and its size detection using cross-sectional segmentation. This segment detection addresses the uncertainty and error in brain image analysis to improve learning precision in different sections and reduce image noises. For instance, compare the previous image output and present the final image output to identify any reform that occurred. Therefore, the maximum pixel representation for radical brain tumors recognition is high, and precision increases.

#### 4.3.4 Precision

**Fig. (9):**
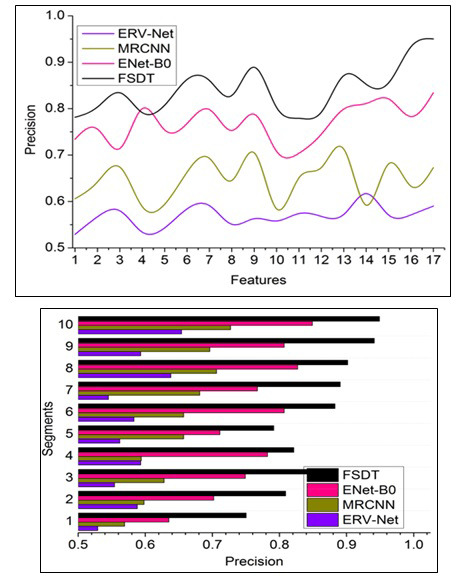
Precision comparisons.

Fig. (**9**) compares the precision of the proposed FSDT with other models. This proposed technique achieves high learning precision for handling sensitive medical images based on pixel distribution analysis with the extracted features aided for accurate tumor detection (Fig. **9**). The precision rate can be calculated with the following formula:

**Table:** 

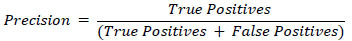

The complexity in identifying tumors and tumors in the brain image is due to minimum and maximum pixel representations operated independently using neural networks. Therefore, brain image analysis is improved, thereby increasing the detection ratio. For this sequence, the pixel distribution is classified through the CNN process, whereas the feature distribution identifies some errors due to classifying such representation. Therefore, the pixel representation is separated to identify accurate tumor detection; the segment detection is processed for better findings. The sequential pixel representation with maximum accuracy for the different sections is used to identify the brain's minimum and maximum reform of tumors. Therefore, this technique has a high maximum pixel distribution leveraging the detection ratio. This radical brain tumors identification based on accurate min/max pixel representation classification is performed by ML using the previous diagnosis for processing fragmentation and segment detection. Detecting such tumors' presence maximizes the pixel representation so that the maximum accuracy achieves high precision in the proposed technique.

#### 4.3.5 Analysis Time

The analysis error and time is processed using sequential min/max pixel representation analysis for the different sections to identify the tumour's accuracy and size in the human brain through feature extraction, illustrated in Fig. (**10**). This proposed technique satisfies less analysis time by computing the fragmentation using cross-sectional segmentation for classifying the pixel distribution for different sections based on the tumor stage, occurrence and its previous impact are used for accurately identifying brain tumor for recovering from this problem. In this brain tumor or tumor detection based on segment detection and feature distribution, the condition *MF*{*UN_C_*(*MRI_images__N_*, C*PD*(*MRI_images__N_*))} is estimated to improve learning precision. The classification errors and noises in the input image are mitigated through feature extraction until maximum accuracy, wherein the different section observation is preceded using Eq. (**4**-**8a**, and **8b**) computations. This proposed technique processes tumor recognition and reform identification to improve the training rate. Based on this sequential pixel representation, the brain image analysis time is less than other factors in this technique. The comparison summary for the features and segments is presented in Tables **2** and **3**.

#### 4.3.6 Recall

Recall assesses the percentage of genuine positive tests that are true positives plus false negatives out of the total true positive instances of properly detected tumors. It measures how well the system can identify tumors when they are visible in the photos. The recall metric is calculated with the formula given below:

**Table:** 

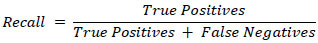

For precise medical situations wherein missing a tumor diagnosis is undesirable, high recall denotes a decreased rate of false negatives. Early diagnosis is essential for prompt intervention and treatment in the discovery of brain tumors. A higher percentage of actual tumors are accurately diagnosed when a recall is high, which increases the likelihood that a tumor will be discovered early. Patient outcomes are directly impacted by a brain tumor detection system's sensitivity. A system with a high recall rate can assist medical professionals in spotting tumors early on when available treatments might be more successful, thus increasing patients' survival rates and quality of life. Fig. (**11**) illustrates the recall measure based on several features and segment-related comparison evaluation.

#### 4.3.7 F1-Score

The F1-score, a statistic, balances precision and recall. It evaluates the relationship between these two criteria and accounts for both positive results and false negatives, presenting a single score using the given formula:

**Table:** 

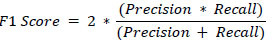

When attempting to achieve a compromise between minimizing inaccurate results and false negatives, the F1-score is particularly helpful. It assists in assessing the overall model's performance. High F1 scores are crucial for fostering confidence in the medical community's usage of computer-aided diagnosis technologies. Clinicians and radiologists can make more informed judgements about patient treatment with the help of these tools. By lowering the likelihood of both a missed diagnosis (missing tumors) and overdiagnosis (false alarms), high F1 scores contribute to better patient care. When it comes to applications in healthcare, wherein the outcomes of patients are at risk, this balance is especially important. Fig. (**12**) illustrates the F1-Score based on several features and segment-related comparison evaluation.

The comparison summary for the features and segments is presented in Tables **2** and **3**.

The proposed technique achieves 10.45% high accuracy, 14.12% high training rate, 9.78% high analysis ratio, 12.56% high precision, F1-score of 2.13%, high recall of 13.3% and 10.78% less analysis time.

The proposed technique achieves 10% high accuracy, 11.28% high training rate, 10.88% high analysis ratio, 10.28% high precision, a high F1-score of 13%, recall of 12.94% and 11.46% less analysis time.

Due to limited time and computational resources, the number of training repetitions in iterations is limited to 800 counts. Extending training beyond a certain iteration can risk overfitting the model to the training data; hence, limiting the iteration count to 800 is necessary. Limiting the training iterations helps prevent overfitting and ensures that the proposed FSDT model generalizes well to unseen data. The findings demonstrate improved accuracy with 10.45% on features and 10% on segments compared to existing approaches. Stable loss and accuracy measures suggest that the model could've reached an acceptable degree of convergence around 800 iterations. It's possible that additional training won't result in appreciable performance enhancements. For deep learning models to converge, which occurs after an adequate number of training iterations around 800 with sufficient training of data source from [35] with a limited quantity of information, the model's weights must alter to minimize the loss function. Convergence can be influenced by the quality of training data and evaluated in the comparison scenario.

### 4.4 Performance Analysis

Analyzing input MRI images thoroughly and extracting information to improve the detection ratio is at the heart of the suggested method for brain tumor identification. These characteristics are broken down by pre-diagnosis and cross-sectional segmentation, which separates the pixel distribution for various parts. The approach successively examines minimum and maximum pixel representations using artificial intelligence and machine learning, efficiently finding uncertainties and faults within the input image. Brain tumor detection is further improved with the use of CNN learning. The method boasts an impressive training rate and combines pre-diagnosis with creating new sections by analyzing brain images at different levels. By concentrating on pixel representation, the segmentation technique can accurately detect tumour size changes, allowing training-based reform. Combining training with the CNN approach drastically reduces analysis time, guaranteeing accurate segment detection. The suggested method, which uses the detection ratio for delicate medical image processing, is characterized by high accuracy. The method achieves optimal accuracy and precision in brain tumor diagnosis by maximizing pixel representation and leveraging past diagnoses for segmentation and segment detection.

Sequential min/max pixel representation analysis defines the method for detecting brain tumours, which reduces analysis time using cross-sectional segmentation. The predicted condition improves training accuracy, processing tumor recognition, and reform identification, leading to a higher training rate. Accurate medical situations rely on recall, which involves evaluating real positive tests. A high recall rate improves patient survival rates and quality of life by aiding early tumor detection and indicating fewer false negatives. We can believe in the suggested method because of the F1-score, a critical statistic that balances recall and precision. With an improved analysis ratio of 9.78%, a 10.45% gain in accuracy, a 14.12% rise in training rate, and a 12.56% increase in precision, the approach shows promise, as seen by a 2.13% increase in F1-score, a 13.3% improvement in recall, and a 10.78% decrease in analysis time. The suggested FSDT model is a significant step forward in brain tumor diagnosis, and limiting training iterations to 800 avoids overfitting, which guarantees strong generalization to new data.

The experimental outcomes show how reliable the suggested FSDT is at identifying brain tumors. Compared to previous approaches, FSDT significantly improves features and segments by 10.45% and 10%, respectively, demonstrating its superior accuracy. Its versatility is shown by its effective training dynamics, which have rates of 96.04% on features and 95.69% on segments. FSDT maximizes the analysis ratio by reducing uncertainty factors, achieving 96.976% on attributes and 97.613% on segments. With high recall (97.1%) and, 95.01% and 94.89% rates, the approach compromises precision and recall. Moreover, FSDT's computational efficiency is demonstrated by its streamlined analysis times, which are 541.835 ms for features and 464.645 ms for segments. Overall, the suggested approach performs better than expected, combining precision, effectiveness, and consistency in identifying brain tumors, and it shows potential for real-world healthcare applications.

The experimental results indicate that the suggested FSDT is computationally economical compared to conventional approaches regarding computational cost. The significant reduction in analysis time suggests that the FSDT yields better performance without compromising computing efficiency.

## CONCLUSION

This research paper has presented an FSDT for identifying brain tumors as a verge for detecting brain tumors. Regardless of the size, the detection accuracy is focused by segregating different cross-sectional views from an input MRI. The proposed technique performs cross-sectional segmentation based on pixel distribution to prevent errors in the detection process. First, the horizontal and vertical pixel distributions are identified using feature extraction and edge separation. The fragmented segments are then identified through cross-sectional extractions, using the uneven distributions to suppress uncertainty. The uncertain pixel distributions across various segments are cumulatively treated for identifying errors. In particular, the minimum and maximum possible representation using the extracted features and mapping pixels are used for classifying the tumor regions. The training based on identified features and their accuracies is performed in the second part of the neural network. This training leverages the precision of identifying new regions through segments or fragment reforms. This is repeated until the highest possible level of saturating accuracy is reached. The proposed technique achieves 10.45% high accuracy, 14.12% high training rate, 9.78% high analysis ratio, 12.56% high precision, and 10.78% less analysis time.

## Figures and Tables

**Fig. (1):**
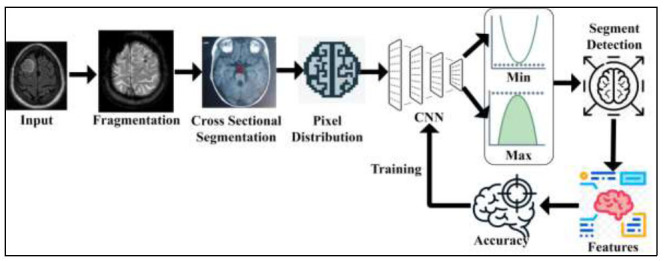
Illustration of the proposed fragmented segment detection technique.

**Fig. (6):**
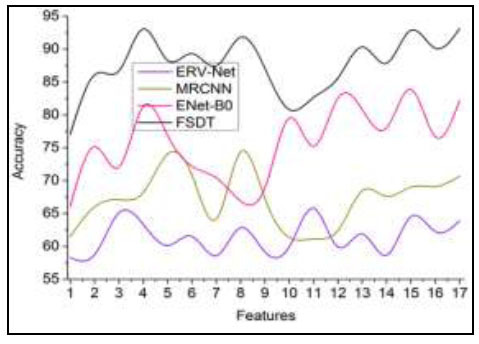
Detection accuracy comparisons.

**Fig. (8):**
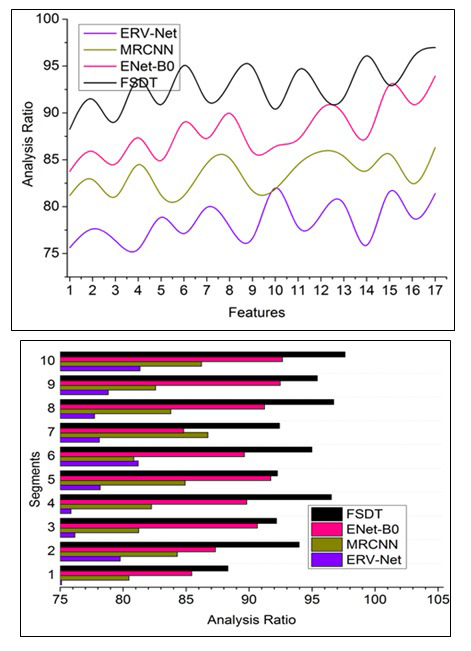
Analysis ratio comparisons.

**Fig. (10):**
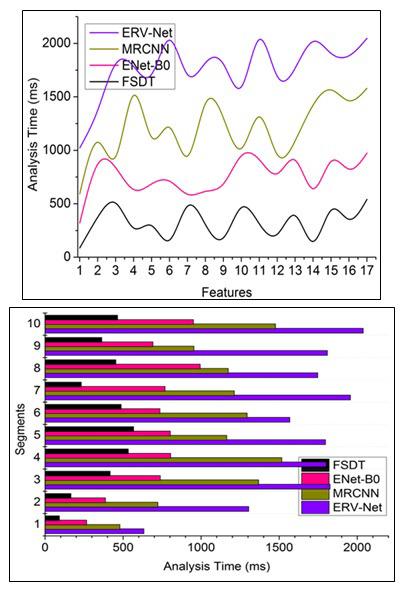
Analysis time comparisons.

**Fig. (11):**
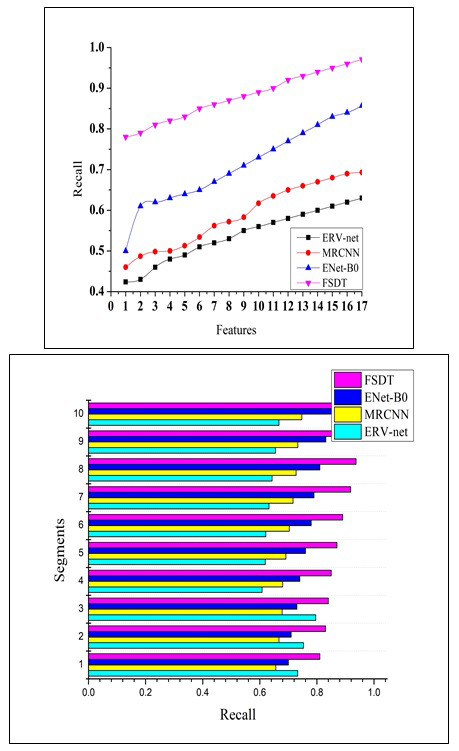
Recall comparison.

**Fig. (12):**
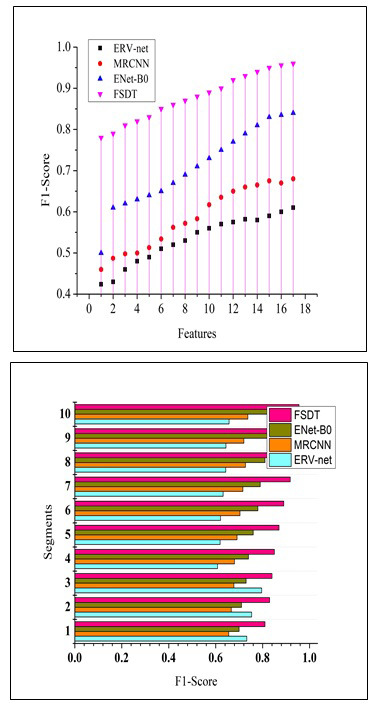
F1-score comparisons.

**Table 1:** Sample input analysis.

Input	C_*FD*_	*ρ_E_*	*C_H_*	*C_V_*
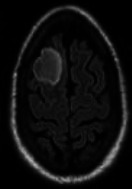	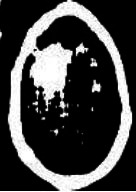	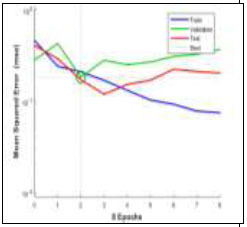	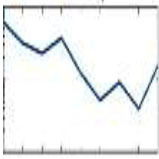	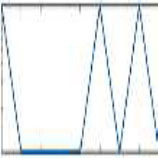
	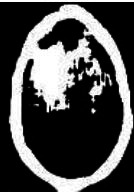	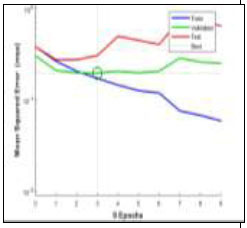	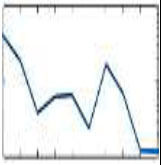	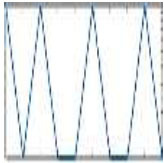
	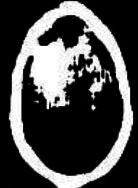	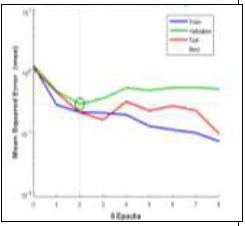	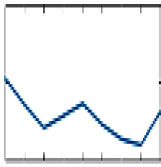	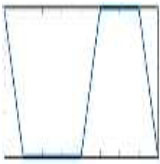
	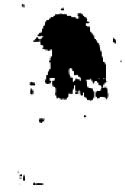	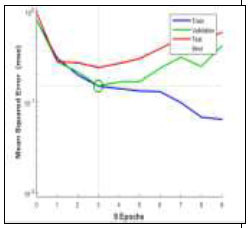	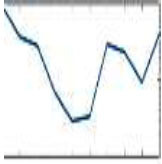	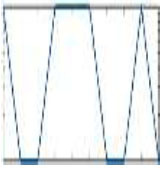

**Table 2:** Comparison summary of features.

**Metrics**	**ERV-Net**	**MRCNN**	**ENet-B0**	**FSDT**
Accuracy	63.865	70.68	82.14	93.128
Training rate	0.576	0.629	0.829	0.9604
Analysis ratio	81.38	86.3	93.92	96.976
Precision	0.59	0.673	0.834	0.9501
Analysis time (ms)	2048.26	1580.63	973.97	541.835
Recall (sensitivity)	0.630	0.693	0.857	0.971
F1-score	0.610	0.680	0.840	0.960

**Table 3:** Comparison summary of segments.

**Metrics**	**ERV-Net**	**MRCNN**	**ENet-B0**	**FSDT**
Accuracy	64.38	72.67	82.75	93.275
Training Rate	0.598	0.721	0.875	0.9569
Analysis Ratio	81.34	86.22	92.65	97.613
Precision	0.654	0.727	0.849	0.9489
Analysis Time (ms)	2037.84	1476.43	950.36	464.645
Recall (Sensitivity)	0.667	0.747	0.858	0.969
F1-Score	0.650	0.730	0.845	0.955

## Data Availability

The data that support the findings of this study are openly accessible on Kaggle at the following link: https://www. kaggle.com/sartajbhuvaji/brain-tumor-classification-mri.

## LIST OF ABBREVIATIONS

FSDT: Feature Segmentation and Detection Technique
CNS: Central nervous system
ML: Machine learning
AI: Artificial intelligence
MRI: Magnetic resonance imaging
ANN: Artificial neural network
ROI: Region of interest
ReLU: Rectified Linear Units
SGD: Stochastic Gradient Descent

## References

[1] Dikici E., Ryu J.L., Demirer M., Bigelow M., White R.D., Slone W., Erdal B.S., Prevedello L.M. (2020). Automated brain metastases detection framework for t1-weighted contrast-enhanced 3D MRI.. IEEE J. Biomed. Health Inform..

[2] Balaha H.M., Hassan A.E.S. (2022). A variate brain tumor segmentation, optimisation, and recognition framework.. Artif. Intell. Rev..

[3] Fang L., Wang X. (2022). Brain tumor segmentation based on the dual-path network of multi-modal MRI images.. Pattern Recognit..

[4] Dweik M., Ferretti R. (2022). Integrating anisotropic filtering, level set methods and convolutional neural networks for fully automatic segmentation of brain tumors in magnetic resonance imaging.. Neuroscience Informatics.

[5] Lu S.L., Liao H.C., Hsu F.M., Liao C.C., Lai F., Xiao F. (2021). The intracranial tumor segmentation challenge: Contour tumors on brain MRI for radiosurgery.. Neuroimage.

[6] Soomro T.A., Zheng L., Afifi A.J., Ali A., Soomro S., Yin M., Gao J. (2022). Image segmentation for mr brain tumor detection using machine learning: A review.. IEEE Rev. Biomed. Eng..

[7] Lei X., Yu X., Chi J., Wang Y., Zhang J., Wu C. (2021). Brain tumor segmentation in MR images using a sparse constrained level set algorithm.. Expert Syst. Appl..

[8] Huang Z., Zou S., Wang G., Chen Z., Shen H., Wang H., Zhang N., Zhang L., Yang F., Wang H., Liang D., Niu T., Zhu X., Hu Z. (2022). ISA-Net: Improved spatial attention network for PET-CT tumor segmentation.. Comput. Methods Programs Biomed..

[9] Hu J., Gu X., Gu X. (2022). Mutual ensemble learning for brain tumor segmentation.. Neurocomputing.

[10] Habib H., Amin R., Ahmed B., Hannan A. (2022). Hybrid algorithms for brain tumor segmentation, classification and feature extraction.. J. Ambient Intell. Humaniz. Comput..

[11] Jiang M., Zhai F., Kong J. (2021). A novel deep learning model DDU-net using edge features to enhance brain tumor segmentation on MR images.. Artif. Intell. Med..

[12] Ma Q., Zhou S., Li C., Liu F., Liu Y., Hou M., Zhang Y. (2022). DGRUnit: Dual graph reasoning unit for brain tumor segmentation.. Comput. Biol. Med..

[13] Zhang Y., Lu Y., Chen W., Chang Y., Gu H., Yu B. (2021). MSMANet: A multi-scale mesh aggregation network for brain tumor segmentation.. Appl. Soft Comput..

[14] Saleem H., Shahid A.R., Raza B. (2021). Visual interpretability in 3D brain tumor segmentation network.. Comput. Biol. Med..

[15] Liu Z., Tong L., Chen L., Jiang Z., Zhou F., Zhang Q., Zhou H. (2022). Deep learning based brain tumor segmentation: A survey.. Complex & Intelligent Systems.

[16] Alqazzaz S., Sun X., Nokes L.D.M., Yang H., Yang Y., Xu R., Zhang Y., Yang X. (2022). Combined features in region of interest for brain tumor segmentation.. J. Digit. Imaging.

[17] Shah H.A., Saeed F., Yun S., Park J.H., Paul A., Kang J.M. (2022). A robust approach for brain tumor detection in magnetic resonance images using finetuned efficientNet.. IEEE Access.

[18] Yu W., Kang H., Sun G., Liang S., Li J. (2022). Bio-inspired feature selection in brain disease detection *via* an improved sparrow search algorithm.. IEEE Trans. Instrum. Meas..

[19] Ahmad S., Choudhury P.K. (2022). On the performance of deep transfer learning networks for brain tumor detection using MR images.. IEEE Access.

[20] Ottom M. A., Rahman H. A., Dinov I. D. (2022). Deep learning approach for 2D MRI brain tumor segmentation.. IEEE J Transl Eng Health Med..

[21] Liang J., Yang C., Zeng L. (2022). 3D PSwinBTS: An efficient transformer-based Unet using 3D parallel shifted windows for brain tumor segmentation.. Digit. Signal Process..

[22] Sunsuhi G.S., Albin Jose S. (2022). An adaptive eroded deep convolutional neural network for brain image segmentation and classification using inception resnetV2.. Biomed. Signal Process. Control.

[23] Ullah Z., Usman M., Jeon M., Gwak J. (2022). Cascade multiscale residual attention CNNs with adaptive ROI for automatic brain tumor segmentation.. Inf. Sci..

[24] Zhou X., Li X., Hu K., Zhang Y., Chen Z., Gao X. (2021). ERV-Net: An efficient 3D residual neural network for brain tumor segmentation.. Expert Syst. Appl..

[25] Pei L., Bakas S., Vossough A., Reza S.M.S., Davatzikos C., Iftekharuddin K.M. (2020). Longitudinal brain tumor segmentation prediction in MRI using feature and label fusion.. Biomed. Signal Process. Control.

[26] Sun J., Peng Y., Guo Y., Li D. (2021). Segmentation of the multimodal brain tumor image used the multi-pathway architecture method based on 3D FCN.. Neurocomputing.

[27] Masood M., Nazir T., Nawaz M., Javed A., Iqbal M., Mehmood A. (2021). Brain tumor localization and segmentation using mask RCNN.. Front. Comput. Sci..

[28] Xiong S., Wu G., Fan X., Feng X., Huang Z., Cao W., Zhou X., Ding S., Yu J., Wang L., Shi Z. (2021). MRI-based brain tumor segmentation using FPGA-accelerated neural network.. BMC Bioinformatics.

[29] Islam M.M., Kashem M.A. (2021). Parametric active contour model-based tumor area segmentation from brain MRI images using minimum initial points.. Iran Journal of Computer Science.

[30] Iqbal M.J., Bajwa U.I., Gilanie G., Iftikhar M.A., Anwar M.W. (2022). Automatic brain tumor segmentation from magnetic resonance images using superpixel-based approach.. Multimedia Tools Appl..

[31] Hossain A., Islam M.T., Rahman T., Chowdhury M.E.H., Tahir A., Kiranyaz S., Mat K., Beng G.K., Soliman M.S. (2023). Brain tumor segmentation and classification from sensor-based portable microwave brain imaging system using lightweight deep learning models.. Biosensors.

[32] Babu K.D., Singh C.S. (2023). Brain tumor segmentation through level based learning model.. Comput. Syst. Sci. Eng..

[33] Ramya D., Lakshmi C. (2024). Design of novel brain tumor segmentation system using hybrid heuristic-aided multiscale self-guided attention mechanism-based adaptive unet+++ with 3D brain MRI images.. Int. J. Pattern Recognit. Artif. Intell..

[34] Sun J., Hu M., Wu X., Tang C., Lahza H., Wang S., Zhang Y. (2024). MVSI-Net: Multi-view attention and multi-scale feature interaction for brain tumor segmentation.. Biomed. Signal Process. Control.

[35] https://www.kaggle.com/sartajbhuvaji/brain-tumor-classification-mri.

